# Translational regulation of periplasmic folding assistants and proteases as a valuable strategy to improve production of translocated recombinant proteins in *Escherichia coli*

**DOI:** 10.1186/s12896-020-00615-0

**Published:** 2020-05-11

**Authors:** Agnieszka Gawin, Helga Ertesvåg, Sine Alise Hartvigsen Hansen, Jostein Malmo, Trygve Brautaset

**Affiliations:** 1 Department of Biotechnology and Food Science, Norwegian University of Science and Technology, Sem Sælandsvei 6-8, N-7491 Trondheim, Norway; 2 Vectron Biosolutions AS, Abels gt 5, N-7030 Trondheim, Norway

**Keywords:** Periplasmic translocation, Recombinant protein production, Translation initiation rate, Ribosome binding site, Genome editing

## Abstract

**Background:**

Advantages of translocation of recombinant proteins to the periplasm in *Escherichia coli* include simplified downstream processing, and improved folding and in vivo activity of the target protein. There are, however, problems encountered in the periplasmic production that can be associated with the incorrect formation of disulfide bonds, incomplete cleavage of the signal peptide, and proteolytic degradation. A common strategy used to overcome these difficulties involves manipulating the cellular levels of proteases and periplasmic folding assistants like chaperones, signal peptide peptidases or thiol-disulfide oxidoreductases. To date, this has been achieved by plasmid-based over-expression or knockouts of the relevant genes.

**Results:**

We changed the translation efficiencies of five native *E. coli* proteins, DsbA, DsbB, Skp, SppA, and DegP, by modifying the strength of their ribosome binding sites (RBS). The genomic RBS sequences were replaced with synthetic ones that provided a predicted translation initiation rate. Single- and double-gene mutant strains were created and tested for production of two pharmaceutically relevant proteins, PelB-scFv173–2-5-AP and OmpA-GM-CSF. Almost all the single-gene mutant strains showed improved periplasmic production of at least one of the recombinant proteins. No further positive effects were observed when the mutations were combined.

**Conclusions:**

Our findings confirm that our strain engineering approach involving translational regulation of endogenous proteins, in addition to plasmid-based methods, can be used to manipulate the cellular levels of periplasmic folding assistants and proteases to improve the yields of translocated recombinant proteins. The positive effects of SppA overexpression should be further investigated in *E. coli*.

## Background

As *Escherichia coli* does not naturally secrete proteins in high amounts neither to the periplasmic nor the extracellular environment [1, 2], the limited translocation efficiency and periplasmic folding capacity may negatively impact high-level production of translocated heterologous proteins. Basic strategies employed to overcome the bottlenecks in folding of translocated proteins rely on the co-expression of periplasmic chaperones, signal peptide peptidases, and thiol-disulfide oxidoreductases, or on deletions of protease genes [3–5]. Plasmid-based methods for supplementing gene expression are often unstable and may require the use of selectable markers and inducers, which is not favorable for industrial purposes. In addition, only a limited number of genes can be co-expressed with a gene of interest and the level of over-expression may exceed a desired threshold, posing an unnecessary metabolic burden [6]. An attractive alternative is rational genome engineering that provides broader generality and leads to construction of plasmid- and marker-less strains with improved capacity for production of translocated recombinant proteins.

Recombinant proteins of eukaryotic origin often carry disulfide bonds, which are formed post-translationally by the oxidation of thiol groups between two cysteine residues. In the reducing environment of the cytoplasm of *E. coli*, disulfide bonds cannot form or are immediately reduced [7]. In turn, in the oxidizing periplasm, introduction of disulfide bonds is catalyzed by the thiol-disulfide oxidoreductase DsbA [8]. After receiving two electrons from a pair of cysteines, DsbA is reoxidized by the inner membrane protein DsbB [9]. It has previously been demonstrated that simultaneous over-expression of DsbA and DsbB from a helper plasmid gives positive effects on production of active horseradish peroxidase (HRP) [10] and on soluble expression of a single-chain variable antibody fragment (scFv) [11].

Solubility of periplasmic proteins can also be increased by the co-expression of molecular chaperones such as Skp. Primary, Skp interacts with outer membrane proteins as they are released from the Sec translocon and assists in their folding and membrane insertion [12]. Together with FkpA, Skp exhibits the widest substrate specificity known among the cellular chaperones [13]. Overexpression of this protein has been reported to enhance soluble yield and antigen binding affinity of a recombinant scFv protein [14].

Another important step in folding and maturation of translocated proteins is cleavage of the signal peptide. The fusion of suitable signal peptides allows for the translocation of precursor proteins through the respective secretion system in the cytoplasmic membrane [15]. Protease IV (SppA) is one of the membrane-bound signal peptide peptidases that initiate proteolytic removal of the signal peptide upon translocation [16, 17]. Recently, it has been shown that over-expression of SppA in *Bacillus licheniformis* strain BL10 resulted in increased recombinant production of nattokinase and α-amylase [18]. Although the SppA homologs from *E. coli* and *B. licheniformis* differ in terms of size and catalytic mechanism, their function remains the same [19, 20]. To our knowledge, no study has been conducted on the effect of native SppA over-expression on production of translocated recombinant proteins in *E. coli*.

Apart from the cleavage of a specific peptide bond, proteolytic activity in the periplasm may involve complete degradation of the protein of interest. The major housekeeping protease responsible for the removal of misfolded and aggregated proteins from the inner-membrane and periplasmic space in *E.coli* is DegP [21]. It has been suggested that DegP null hosts should be routinely used for high-level production of heterologous proteins [13]. In fact, strains deficient in DegP have frequently been constructed, and this resulted in improved yield of proteolytically sensitive peptides such as scFv and Fab antibody fragments [22–24].

In this study, the effect of changing the chromosomal translation initiation rates of DsbA, DsbB, Skp, SppA, and DegP on the production of translocated recombinant proteins in *E. coli* RV308 was investigated. To obtain the desired translation efficiencies, we replaced the native ribosome-binding site (RBS) sequences upstream of the open reading frames with designed synthetic RBS variants. The engineered strains were subsequently tested for production of two pharmaceutically relevant model proteins, the single-chain antibody fragment 173–2-5 fused to alkaline phosphatase and equipped with the PelB signal peptide (PelB-scFv173–2-5-AP) [25], and granulocyte-macrophage colony-stimulating factor with the OmpA signal sequence attached (OmpA-GM-CSF) [26] (sequences of the genes encoding the model proteins are given in Table S3). This allowed us to investigate the effect of the mutations on the production of different heterologous proteins fused to two different signal sequences. The single-chain variable fragment scFv173–2-5 is a 27.2 kDa protein containing four intramolecular disulfide bonds, while recombinant human GM-CSF has two disulfide bonds and a molecular weight of 14.6 kDa. Both PelB-scFv173–2-5-AP (77.2 kDa) and OmpA-GM-CSF (17.3 kDa) have previously been used as model proteins to study periplasmic protein yields [25, 27].

## Results

### Design of RBS sequences and construction of the mutant strains

The translation initiation rates (TIRs) of the native RBS sequences for DsbA, DsbB, Skp, SppA, and DegP were predicted with the RBS Calculator developed to enable the design of 5′- untranslated regions (5′-UTRs) for desired expression level [28, 29], and are listed in Table 1. In order to determine desired TIRs of the synthetic RBS variants, we investigated the previously reported levels of translation efficiency that can be achieved by co-expressing a gene of interest from a plasmid. Expression systems that are commonly used for such purpose are the arabinose inducible *P*_*BAD*_ [10], and the IPTG inducible *lac* promoter [14]. As previously shown, the 5′- UTRs of these promoter systems provide relatively high translation efficiencies ranging from 11,408 to 120,064 arbitrary units (au) for the *P*_*BAD*_, and from 6897 to 33,626 au for the *lac* promoter, for different gene products [25]. Instead of co-expressing the target genes, we decided to evaluate how changing the translation rate of the selected native proteins affects periplasmic production of two model recombinant proteins. Synthetic RBS sequences were again designed by the RBS Calculator aiming for an up to 10-fold change in translation. The TIR values for DsbA, DsbB, Skp, SppA were upregulated, while translation efficiency for DegP was 7.5-fold downregulated (Table 1).

**Table 1:** In silico analysis of TIR values and RBS sequences (denoted in capital letters) with adjacent genomic regions

Gene	Native TIR (au)	Regulated TIR (au)	Native RBS (5′ – 3′)	Synthetic RBS (5′ – 3′) ^a^
*dsbA*	38,952	387,845	//−tgtaTTAATCGGAGAGAGTAGATCatga−//	//−gatcAACTAAGGAGGCTTATTatga−//
*dsbB*	158	1783	//−tatgCATATTGCAGGGAAATGATTatgt−//	//−gattGCGCACACCGAAGTTGGAATACGATTGatgt−//
*skp*	19,377	80,140	//−ccggTGCAAATGGGATGGTAAGGAGTTTATTgtga−//	//−ccggCAGTATATACGTCTAACACTTAGGGGGAATATTAtga−//
*sppA*	4205	19,838	//−tattGCGCCTGTGACAGGTGTGACCTTAAGTTGGGAGAATACatgc−//	//−tattCAAACGTACCCCTTAATTATACCTAACGAGGAGAAACTatgc−//
*degP*	2139	286	//−ttttGCGTTATCTGTTAATCGAGACTGAAATACatga−//	//−ttttGAGTAACGCCACTGATCGAAATTGAGGAAGtga−//

^a^ Desired TIR values for *skp* and *degP* were obtained by RBS replacement and substitution of the first base (underlined above) of a start codon; GTG → ATG and ATG → GTG, respectively

The synthetic RBS sequences were introduced to the genome of the industrially relevant *E. coli* strain RV308 using CRMAGE [30]. For this purpose, gRNA sequences for CRISPR/Cas9 negative selection were designed in a way to fully or partially cover a region of the native RBS sequences. In the cases of *sppA* and *skp*, PAM motifs were identified within the RBS regions to be mutated and could be removed by introducing synthetic RBS sequences to allow for the Cas9-based negative selection against the wild-type sequences. For the rest of the genes, PAM sites were identified close to the mutated region and disrupted by a secondary silent mutation (Table S2). After a CRMAGE round, randomly picked colonies carrying one of the desired mutations were selected with colony PCR. In order to study a combined effect of some of the mutations, double-gene mutants RV308(*dsbA*^*rbs*^*dsbB*^*rbs*^), RV308(*degP*^*rbs*^*sppA*^*rbs*^), RV308(*degP*^*rbs*^*skp*^*rbs*^), RV308(*sppA*^*rbs*^*skp*^*rbs*^) were constructed using a second CRMAGE round. All RBS replacements were confirmed with DNA sequencing. Each of the plasmids used for CRMAGE, except the pMAZ-SK_x that is equipped with a self-killing mechanism, was cured with the pFREE-based system [31]. Prior to expression studies, plasmid-free mutant strains were transformed either with pSB-M1s carrying gene encoding PelB-scFv173–2-5-phoA fusion protein [25] or pGM29ompA carrying gene encoding OmpA-GM-CSF [26].

### The *dsbA* mutation displays positive effects on periplasmic levels of both model proteins and for the *dsbB* mutant strain the effect is protein-dependent

**Fig. 1:**
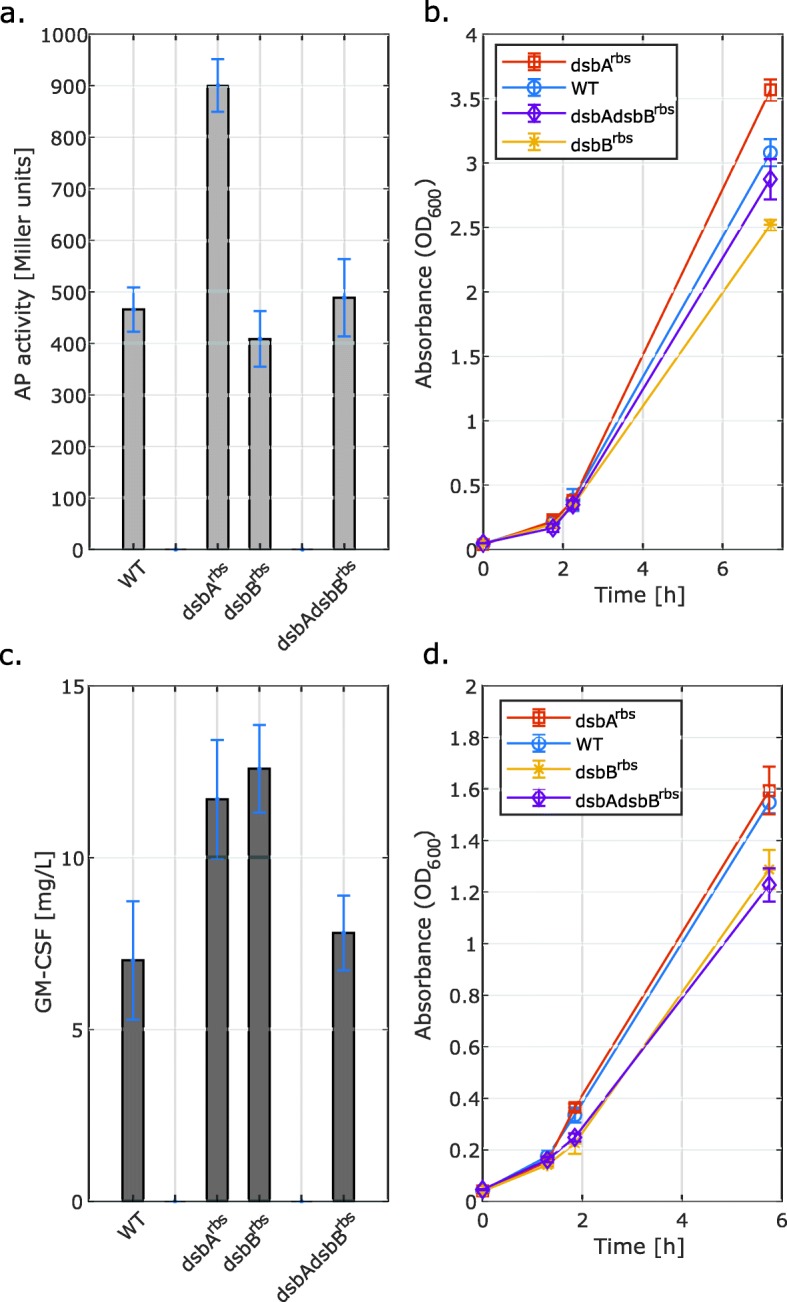
Comparison of the wild-type *E. coli* strain RV308 (WT) and mutant strains RV308(*dsbA*^*rbs*^), RV308(*dsbB*^*rbs*^), and RV308(*dsbA*^*rbs*^*dsbB*^*rbs*^) with regulated translation rate of components of the disulfide bond formation mechanism. The panels show AP activity **a** and concentration of GM-CSF **c** under induced conditions and the corresponding growth curves: pSB-M1s **b**, pGM29ompA **d**. Following 2 h incubation, the XylS/*Pm*-mediated protein expression was induced (OD600 ~ 0.3–0.5) by adding m-toluic acid to a final concentration of 1 mM. The AP activity and GM-CSF concentration were measured in the periplasmic fraction of cells harvested 4 h (pGM29ompA) and 5 h (pSB-M1s) post induction. The data presented are the averages of three biological replica with the standard deviation indicated. The AP activity and GM-CSF concentration data were normalized against the total protein content measured in the periplasmic fraction

As DsbA and DsbB act together as a part of the oxidation system for disulfide-bond formation, the RV308(*dsbA*^*rbs*^) and RV308(*dsbB*^*rbs*^) single-gene mutants but also the RV308(*dsbA*^*rbs*^*dsbB*^*rbs*^) double-gene mutant were created. All three strains were tested for production of PelB-scFv173–2-5-AP fusion protein and OmpA-GM-CSF. Production of translocated scFv173–2-5-AP was monitored as alkaline phosphatase (AP) activity in periplasmic lysates, which means that only functional protein was measured, and that the AP protein was successfully translocated and folded in the periplasmic space. In the case of OmpA-GM-CSF, the GM-CSF protein concentration in periplasmic lysates was measured using ELISA. Periplasmic and cytoplasmic protein samples of all strains were also run on SDS-PAGE gel (Figs. S2 and S3). A slight improvement of growth rate and a 1.9- (*P* < 0.001, Table S1) and 1.7-fold increase (*P* < 0.05, Table S1) in AP activity and GM-CSF concentration, respectively, were detected when the fusion protein was expressed in the *dsbA* mutant strain compared to the wild-type RV308 (Fig. 1). In turn, the upregulation of the translation initiation rate of DsbB, both individually and in combination with DsbA, had a negative impact on cell growth with accompanying lack of change or even a slight decrease of AP activity (Fig. 1a and b). However, this effect was protein-dependent, as in the case of GM-CSF, a 1.8-fold increase (*P* < 0.05, Table S1) in concentration of the protein was detected for the RV308(*dsbB*^*rbs*^) single-gene mutant strain (Fig. 1c). Reduced growth triggered by the upregulated translation of native DsbB was observed also during production of OmpA-GM-CSF (Fig. 1d).

### Mutations of the RBS regions of *skp, sppA* and *degP* resulted in statistically significant increase of periplasmic levels of one or both of the model proteins

**Fig. 2:**
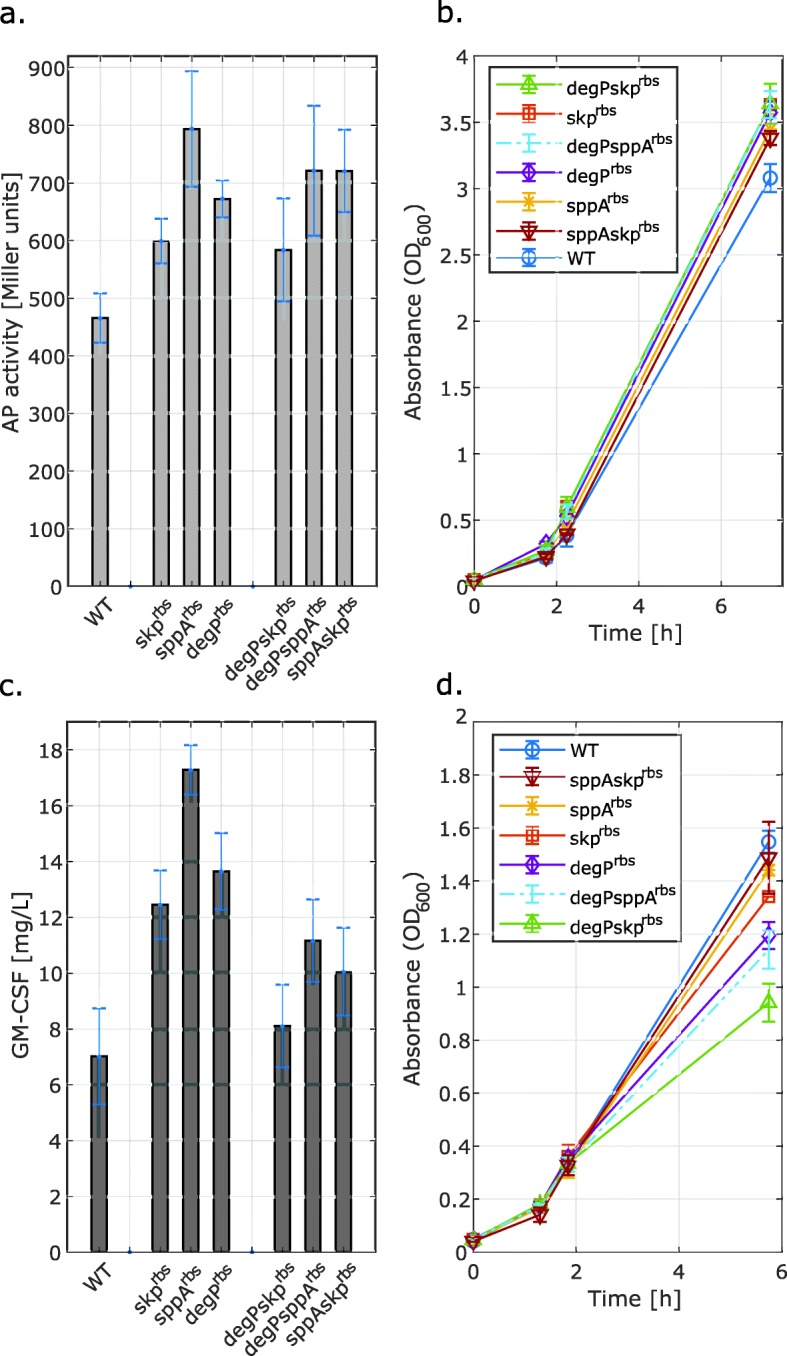
Comparison of the wild-type *E. coli* strain RV308 (WT) and mutant strains RV308*(skp*^*rbs*^), RV308(*sppA*^*rbs*^)*,* and RV308(degP^rbs^) with regulated translation of Skp (folding chaperone), SppA (signal peptidase), and DegP (periplasmic protease). The panels show AP activity **a** and concentration of GM-CSF **c** under induced conditions and the corresponding growth curves: pSB-M1s **b**, pGM29ompA **d**. Following 2 h incubation, the XylS/Pm-mediated protein expression was induced (OD600 ~ 0.3–0.5) by adding m-toluic acid to a final concentration of 1 mM. The AP activity and GM-CSF concentration were measured in the periplasmic fraction of cells harvested 4 h (pGM29ompA) and 5 h (pSB-M1s) post induction. The data presented are the averages of three biological replica with the standard deviation indicated. The AP activity and GM-CSF concentration data were normalized against the total protein content measured in the periplasmic fraction

Unlike the mutations of the components of disulfide bond formation mechanism, the RV308(*skp*^*rbs*^) RV308(*sppA*^*rbs*^) and RV308(*degP*^*rbs*^) single-gene mutants showed a similar overall pattern in protein activity and concentration of scFv173–2-5-AP and GM-CSF, respectively (Fig. 2a, c). For the strain with a regulated translation of SppA protease, a 1.7-fold increase (*P* < 0.001, Table S1) in AP activity and a 2.5-fold increase (*P* < 0.001, Table S1) in GM-CSF concentration were observed. A positive effect of TIR upregulation was achieved also for the RV308(*skp*^*rbs*^) mutant strain, which showed 1.8-fold increase (*P* < 0.05, Table S1) in GM-CSF concentration (Fig. 2c). DegP was the only studied protein with attenuated TIR, and the strain deficient in this protease showed 2-fold higher (*P* < 0.001, Table S1) concentration of GM-CSF (Fig. 2c). Although Fig. 2a might indicate that both the RV308(*skp*^*rbs*^) and RV308(*degP*^*rbs*^) single-gene mutants showed improved AP activity, these effects turned out to be not significant (*P* ≥ 0.05). All the single-gene mutations promoted the growth of cells during the expression of PelB-scFv173–2-5-AP, while the growth of mutant strains producing OmpA-GM-CSF, especially the RV308(*degP*^*rbs*^), decreased considerably compared to the wild-type strain (Fig. 2b, d).

Based on the results showing improved productivity of all three single-gene mutants, we decided to investigate the potential additive effects of the mutations. Therefore, the RV308(*degP*^*rbs*^*sppA*^*rbs*^), RV308(*degP*^*rbs*^*skp*^*rbs*^) and RV308(*sppA*^*rbs*^*skp*^*rbs*^) double-gene mutant strains were created. Although for the RV308(*sppA*^*rbs*^*skp*^*rbs*^) and RV308(*degP*^*rbs*^*sppA*^*rbs*^) the scFv173–2-5-AP activity and GM-CSF concentration remained significantly higher in comparison to the wild-type strain, introduction of any of the additional mutations was not effective. Instead, by replacing the native RBS sequence upstream of e.g. *skp* gene in the *degP* mutant, the final GM-CSF concentration was reduced 1.7-fold. For production of GM-CSF, the presence of a second mutation in the *degP* strain had further negative impact on the growth rate (Fig. 2d). Still, even when the GM-CSF data was calculated per OD unit (Fig. S1), similar trends were obtained for all constructed strains.

## Discussion

Some approaches in the development of *E. coli* strains for the secretion of pharmaceutical proteins to the periplasm were recently reviewed by Castineiras et al. [32]. To date, host strain engineering in this field has mostly been based on minimizing protease activity through target gene knockouts, or on random mutagenesis leading to the directed evolution of the secretion apparatus or increased membrane permeability [33]. However, it is worth noting that Browning et al. [34] recently developed the TatExpress strains, where transcriptional control of the chromosomal *tatABCD* operon allowed for improved recombinant periplasmic production of human growth hormone (hGH) and a scFv. While modulating the transcriptional efficiency enables expression of multiple genes at the desired level, balanced protein expression also requires rational optimizing of post-transcriptional processes [28, 35]. Here, we demonstrate that optimization of chromosomal protein synthesis via translational control based on RBS engineering can be used to regulate functional properties of bacterial strains. Although we did not investigate the expression levels of the endogenous proteins that were targeted in this work, we evaluated how the predicted change of translation initiation rate affects the production of two translocated recombinant proteins. Our results indicate that most of the single-gene mutations facilitated the production of at least one of the translocated heterologous proteins. The best performing strain, in terms of production of both model proteins, was the single-gene mutant with upregulated translation rate of SppA. To our knowledge, previous studies on the effect of over-expressing this signal peptidase on production of translocated recombinant proteins have only been conducted in *B. licheniformis* [18], not in *E. coli*.

As the introduction of any of the additional mutations was not effective, our trial-and-error approach should rather be extended by a selective optimization involving a construction of RBS libraries and high-throughput screening of the most productive strains [36, 37]. This is especially relevant in the case of DsbA and DsbB as simultaneous overexpression of these proteins has previously been shown to give positive effects on periplasmic recombinant protein production [10]. Interestingly, the RV308(*dsbA*^*rbs*^) mutant strain showed highest activity of AP among all studied strains, while in the case of the RV308(*dsbA*^*rbs*^*dsbB*^*rbs*^), the AP activity and GM-CSF concentration were not increased. This may indicate that upregulation of TIR for both DsbA and DsbB accompanied by the recombinant protein production could overburden the cells. In addition, as seen on the example of the RV308(*dsbB*^*rbs*^), the outcome of the mutations might be dependent on the size and structure of the translocated protein, but also on the choice of the signal peptide, which was shown to strongly influence the total expression levels of different proteins [26, 38]. It was previously demonstrated that *dsbB* mutations affect the redox status of the periplasm [39] and this could explain the observed growth inhibition of the RV308(*dsbB*^*rbs*^) and RV308(*dsbA*^*rbs*^*dsbB*^*rbs*^) mutants. The growth could also be affected by incorrect folding of the native and/or model proteins.

As previously reported [40], the positive effects of chaperone overexpression on recombinant protein production can be masked when the level of synthesis of chaperones imposes a metabolic burden itself. This could be demonstrated in the RV308(*sppA*^*rbs*^*skp*^*rbs*^) strain producing OmpA-GM-CSF. The levels of this heterologous protein produced by the RV308(*sppA*^*rbs*^) single-gene mutant were already high relative to other strains, and we observed the slight growth inhibition that does not occur upon induction of PelB-scFv173–2-5-AP expression. Introducing additional mutation and upregulation of the translation of Skp had no further positive outcome on soluble protein levels and could have overburdened the metabolomic capacities. This, however, does not explain a negative effect of combining the *skp* or *sppA* mutations with the *degP* mutation as the TIR value for this chromosomal target was downregulated. The proteolytic activity of DegP has been found to be essential for effective removal of cell-membrane-damaging misfolded proteins at heat-shock temperatures [41]. The over-expression of recombinant and/or chromosomal proteins could therefore be particularly toxic in DegP deficient strains. However, the RV308(*degP*^*rbs*^) single-gene mutant showed relatively high enhancement GM-CSF concentration. The DegP mutant created by Chen et al. [22] also produced higher levels of heavy and light chains and F (ab´)_2_ antibody fragments, but the range of increase did not exceed 1.7-fold compared to the wild-type *E. coli* W3110, and the higher-level production required a triple-mutant of *degP*, *prc* and *spr*. It was previously demonstrated that co-expression of Skp from a helper plasmid may result in up to 10-fold enhancement of scFvD1.3 production [42]. This indicate that more optimal rates of the native Skp synthesis should potentially be identified, as these may vary both with the recombinant protein and with its expression level.

## Conclusions

In this study, we improved the periplasmic production of two different model recombinant proteins by creating *E. coli* RV308 mutant strains. For this purpose, we designed synthetic ribosome binding sites to regulate the translational efficiency of five chromosomally encoded proteins, DsbA, DsbB, Skp, SppA, and DegP that function as protein folding assistants or proteases. The presented strain engineering based on translational control of native proteins is a novel approach in improving heterologous production compared to commonly used knockouts and plasmid-based methods for supplementing gene expression. The data presented here demonstrate the beneficial effects of most of the introduced mutations and that these effects are abolished when the mutations are combined. This indicates a need to balance the periplasmic chaperone and protease levels when modifying their endogenous expression levels. We report that the SppA signal peptidase should be considered a relevant candidate for further studies on optimization of production signal peptide carrying recombinant proteins in *E. coli*.

## Methods

### Bacterial strains and their cultivation

Strain *E. coli* DH5α (Bethesda Research Laboratories) was used for pMAZ-SK_x plasmids cloning. *E. coli* RV308 (ATCC 31608) served as a protein expression host and was mutagenized using the CRMAGE approach (see below). Cells were routinely grown at 37 °C with 225 rpm shaking in L broth (10 g/L tryptone, 5 g/L yeast extract, and 5 g/L NaCl) or on L agar plates (L broth containing 15 g/L agar). For plasmid selection, ampicillin, spectinomycin, and kanamycin were used at concentrations 100 μg/ml, 100 μg/ml, and 50 μg/ml, respectively. For comparative analysis of mutant and wild-type strains, all strains were transformed with pSB-M1s [25] or pGM29ompA [26]. The pSB-M1s and pGM29ompA expression vectors are based on the RK2 replicon [43] with the heterologous model genes placed under control of the XylS/*Pm* promoter system previously reviewed in details [44]. The pSB-M1s and pGM29ompA plasmids carry kanamycin and ampicillin resistance genes, respectively.

In order to determinate protein production by the strains, the recombinant cells carrying the respective plasmids were grown overnight in 5 ml of a liquid medium with appropriate antibiotics. Afterwards, 20 ml of the fresh medium with the adequate antibiotic was inoculated with the overnight culture to an initial OD_600_ of 0.05. Following around 2 h incubation in 250-ml shake flasks at 30 °C with 225 rpm shaking, the XylS/*Pm*-mediated protein expression was induced (OD_600_ ~ 0.3–0.5) by adding m-toluic acid to a final concentration of 1 mM. The incubation was continued at 30 °C for 5 h to induce the expression of scFv173–2-5-AP, and for 4 h to induce the expression of GM-CSF. After this time, the cells were harvested, and periplasmic protein extraction was carried out.

### Genome engineering and plasmid curing

Plasmids used in this study are presented in Table 2. The sequences of CRMAGE oligos, gRNAs and primers are listed in Table S2. The CRMAGE oligos were designed to contain the desired RBS region flanked by short homology arms (~ 40–90 bp) complementary to the genomic region adjacent to the mutation site. The CRMAGE oligos were synthesized as single-stranded DNA sequences (Biolegio BV, Nijmegen, The Netherlands). The pMAZ-SK plasmid was used for the negative selection to increase the efficiency of CRMAGE. The plasmid carries sgRNA specific to the non-mutated genome sequence that is intended to be cleaved by Cas9 [30]. In order to change the target sequence of pMAZ-SK plasmid, the pMAZ-SK backbone was amplified with the primer pair Gibson_ pMAZ-SK backbone_F/R, and the gRNAs were synthesized as two complementary single-stranded oligos with overlaps matching the adjacent regions of the plasmid backbone and annealed. The pMAZ-SK_x plasmids were created by Gibson assembly [46] of annealed gRNAs and the amplified pMAZ-SK backbone. The CRMAGE procedure started with transforming the wild-type *E. coli* RV308 strain with pZS4Int-tetR and pMA7CR_2.0. The pMA7CR_2.0 plasmid carries genes encoding λ Red recombinase and Cas9, while plasmid pZS4Int-tetR contains a gene encoding *Tet*R transcriptional factor needed for repression of the *P*_*Ltet*_ promoter (controlling the expression of Cas9) in the absence of the inducer.

**Table 2:** Plasmids used in this study

Name	Key features	Source
pZS4Int-tetR	Gene encoding the *Tet*R repressor under control of the constitutive P_N25_ promoter, SC101 ori, Sp^r^	[45]
pMA7CR_2.0	λ Red β-protein under control of the L-arabinose inducible *P*_*BAD*_, Cas9 under control of the anhydrotetracycline inducible *P*_*Ltet*_, ColE1 ori, Amp^r^	[30]
pMAZ-SK	sgRNA targeting *galK* under control of the anhydrotetracycline inducible *P*_*Ltet*_, L-rhamnose inducible plasmid self-killing mechanism, ColA ori, Km^r^	[30]
pMAZ-SK_x	pMAZ-SK with gRNA variants targeting x gene: … *dsbA* … *dsbB* … *skp* … *sppA* … *degP*	This study
pFREE	Cas9 under control of the anhydrotetracycline inducible *P*_*Ltet*_, gRNA targeting the most common replicons under control of the L-rhamnose inducible *P*_*rha*_, ColA ori, *tetR*, Km^r^	[31]
pSB-M1s	*pelB-scFv173–2-5-phoA* fusion gene under control of the *m*-toluate inducible XylS/*Pm* promoter system, RK2 replicon, Km^r^	[25]
pGM29ompA	gene encoding GM-CSF with *ompA* signal sequence under control of the *m*-toluate inducible XylS/*Pm* promoter system, RK2 replicon, Amp^r^	[26]

The recombinant cells carrying the two different plasmids were grown overnight in 5 ml of a liquid medium with appropriate antibiotics. Afterward, 15 ml of the fresh medium with adequate antibiotics was inoculated with the overnight culture to an initial OD_600_ of 0.05. Following 2 h incubation (OD_600_ ~ 0.3–0.5) in 250-ml shake flasks at 37 °C with 225 rpm shaking, the *P*_*BAD*_ -mediated expression of λ Red recombinase was induced by adding L-arabinose to a final concentration of 0.2%, followed by incubation at 37 °C with 225 rpm shaking for 15 min. The cells were pelleted by centrifugation and transformed by electroporation with the corresponding pMAZ-SK_x plasmid and CRMAGE oligo as described by Ronda et al. [30]. Ampicillin (selection for pMA7CR_2.0) and spectinomycin (selection for pZS4Int-tetR) were added immediately after transformation and the cells were transferred to a fresh tube (15 ml) and incubated at 37 °C with 225 rpm shaking for 1 h. Then kanamycin (selection for pMAZ-SK_x) was added and the cultures were incubated for additional 2 h at the same conditions. The *P*_*Ltet*_-mediated expression of Cas9 was induced by adding anhydrotetracycline to a final concentration of 200 ng/ml and the cells were left to grow overnight and plated on selective media (10^− 4^ dilution). Colonies carrying desired mutations were identified with colony PCR. All RBS replacements were eventually confirmed with DNA sequencing.

In order to cure the pMAZ-SK_x plasmids, mutant cells were grown overnight in 5 ml of liquid medium in the presence of kanamycin. Afterward, 10 ml of the fresh medium with adequate antibiotics only for selection of pZS4Int-tetR and pMA7CR_2.0 were inoculated with the overnight culture diluted 1:100. The cultures were grown for 4 h and expression of Cas9 and self-killing gRNA was induced by addition of anhydrotetracycline (200 ng/ml) and L-rhamnose (0.2% w/v), respectively. The incubation was continued overnight, and the cells were plated on solid media lacking kanamycin.

Plasmid pFREE was used to cure the pZS4Int-tetR and pMA7CR_2.0 by using the procedure described by Lauritsen et al. [31]. The cells carrying the two different plasmids were grown overnight in 5 ml of a liquid medium with appropriate antibiotics. Afterward, 10 ml of the fresh medium with adequate antibiotics was inoculated with the overnight culture to an initial OD_600_ of 0.05. Following 2 h incubation (OD_600_ ~ 0.3–0.5) in 250-ml shake flasks at 37 °C with 225 rpm shaking, the cells were pelleted and transformed with the pFREE by electroporation as described by Lauritsen et al. [31]. The cells were incubated in 500 μl a liquid medium at 30 °C with 225 rpm shaking for 2 h and transferred to 10 ml of a fresh liquid medium with anhydrotetracycline (200 ng/ml) and L-rhamnose (0.2% w/v) and kanamycin. The growth was continued overnight at 30 °C with 225 rpm shaking and plated on solid agar plates without antibiotics. The loss of all the plasmids was confirmed by testing antibiotic sensitivity of all the strains.

### Purification of periplasmic proteins

Disruption of the *E. coli* outer membrane and recovery of the periplasmic content was achieved by osmotic shock as previously described [47] with the modifications presented by Hong et al. [48]. Cultures were thawed on ice, harvested by centrifugation (7000 x g, 10 min, 4 °C) and resuspended in 1 ml of ice-cold 20% w/v sucrose-0.03 M Tris-HCl (pH 8.0). The suspension was treated with 0.25 ml of 5 mM disodium EDTA (pH 8.0) and mixed on a rotating shaker (Multi Rotator, 80 rpm) for 10 min at room temperature. After that, the mixture was centrifuged (13,000 x g, 10 min, 4 °C) and resuspended in an equal volume (1.25 ml) of ice-cold water. The suspension was mixed in an ice bath on a rotary shaker for 10 min and centrifuged (13,000 x g, 10 min, 4 °C). The supernatant containing the periplasmic fraction was removed and stored at − 20 °C until the AP activity assay and ELISA could be completed. The pellet containing the cytoplasmic contents was stored at − 20 °C. The total protein content in the periplasmic fraction was measured with a NanoDrop spectrophotometer (A_280_).

### AP activity assay and ELISA

Detection of translocated soluble scFv173–2-5-AP and GM-CSF proteins was done by AP activity assay and ELISA, respectively. The AP activity was measured by following the procedure described previously [49] with slight modifications. 25 μl of the periplasmic fraction was mixed with 100 μl of 0.001 M *p*-nitrophenyl phosphate-1 M Tris-acetate (pH 8.0). After incubation at 37 °C for 30 min, the reaction was stopped by addition of 25 μl of 0.75 M K_2_HPO_4_ (pH 7.0). The sample was diluted 1:10 with 1 M Tris-acetate (pH 8.0) in a microtiter plate and absorbance was read at 420 nm and 550 nm. Units of phosphatase activity was calculated by the Brickman-Beckwith formula [50].

The concentration of GM-CSF in the periplasmic fractions was quantified by using Human GM-CSF ELISA Kit (Diaclone Research, Besançon, France) in accordance with the manufacturer’s instructions.

### SDS-PAGE analysis

For SDS-PAGE analysis, the pellets containing the cytoplasmic contents were treated with CelLytic™ B Cell Lysis Reagent (Sigma-Aldrich). An additional 50 units/mL benzonase nuclease (Sigma-Aldrich) was used for all samples. The samples were incubated for 30 min at room temperature with shaking (100 rpm). Dilutions (5x) of the samples were done with XT MES Running Buffer (Bio-Rad). Each of the samples was combined with XT Sample Buffer 2X (Bio-Rad) and with XT Reducing Agent (Bio-Rad) and incubated at 95 °C for 5 min. Samples (10 μL, 10x diluted) and a ladder (Precision Plus Protein™ Dual Color Standards, Bio-Rad, 5 μL) were loaded on SDS-gel (Criterion™ XT Bis-Tris Precast Gels, 12%, Bio-Rad) for gel electrophoresis (200 V, 45 min), which after end of run was stained with InstantBlue™ Coomassie Protein Stain (Expedeon).

## Supplementary information

**Additional file 1:** Statistical analysis [51]. **Table S1.** Holm-Šídák pairwise multiple comparison of the strains testing the effect of mutations on the production of model proteins. **Figure S1.** The GM-CSF data calculated per OD_600_ unit. **Table S2.** Oligonucleotides used in this study. **Table S3.** DNA sequences of the genes encoding the model proteins used in this study. **Figure S2.** SDS-PAGE of reduced periplasmic and cytoplasmic samples (10x diluted) of scFv173-2-5-AP producing strains. **Figure S3.** SDS-PAGE of reduced periplasmic and cytoplasmic samples (10x diluted) of GM-CSF producing strains.

## Data Availability

The datasets used and/or analysed during the current study are available from the corresponding author on reasonable request.

## Abbreviations

AP: Alkaline phosphatase
ANOVA: Analysis of variance
au: Arbitrary units
hGH: Human growth hormone
HRP: Horseradish peroxidase
OmpA-GM-CSF: Granulocyte-macrophage colony-stimulating factor with the OmpA signal sequence attached
PelB-scFv173–2-5-AP: Single-chain antibody fragment 173–2-5 fused to alkaline phosphatase and equipped with the PelB signal peptide
RBS: Ribosome binding sites
scFv: Single-chain variable antibody fragment
SD: Standard deviation
TIRs: Translation initiation rates
5′-UTRs: 5′- untranslated regions
