# Supplementary material for: Translational regulation of periplasmic folding assistants and proteases as a valuable strategy to improve production of translocated recombinant proteins in Escherichia coli
Source: BMC Biotechnol. 2020 May 11;20:24. doi: 10.1186/s12896-020-00615-0 (PMC7216727; doi:10.1186/s12896-020-00615-0)
Supplement: Supplementary file 1 — Additional file 1: Statistical analysis [51]. Table S1. Holm-Šídák pairwise multiple comparison of the strains testing the effect of mutations on the production of model proteins. Figure S1. The GM-CSF data calculated per OD600 unit. Table S2. Oligonucleotides used in this study. Table S3. DNA sequences of the genes encoding the model proteins used in this study. Figure S2. SDS-PAGE of reduced periplasmic and cytoplasmic samples (10x diluted) of scFv173-2-5-AP producing strains. Figure S3. SDS-PAGE of reduced periplasmic and cytoplasmic samples (10x diluted) of GM-CSF producing strains. [file 12896_2020_615_MOESM1_ESM.docx]

**Additional file 1:**

**Statistical analysis**

The sample standard deviation (SD) was calculated using the “n-1” method (51) according to the equation:

|  | SD = $\sqrt{\frac{\sum_{i=1}^{n} {(x_{i}- \bar{x})}^{2}}{n-1}}$ | (1) |
| --- | --- | --- |

where $n$ is a number of measurements, $x_{i}$ is a value of individual data points, and $\bar{x}$ is a mean of the data points.

The experimental data were analysed by a one-way analysis of variance (ANOVA) followed by a post hoc Holm-Šídák test using SigmaPlot 14.0 software (Systat Software Inc.). The significance was determined using a probability value of *P* < 0.05. The results of pairwise multiple comparison showing significant differences between the studied strains are presented in Table S1.

**Table S1 Holm-Šídák pairwise multiple comparison of the strains testing the effect of mutations on the production of model proteins.** The data passed the Shapiro-Wilk normality test and Brown-Forsythe equal variance test. Only comparisons that revealed significant difference (P < 0.05) between the strains are shown in the table. DM, difference of means; t, t-value.

| **Strain comparison** | **DM** | ***t*** | ***P*** |
| --- | --- | --- | --- |
| **PelB-scFv173-2-5-AP producing strains:** | | | |
| dsbA vs. dsbB | 491.999 | 8.402 | <0.001 |
| dsbA vs. RV308 WT | 435.047 | 7.429 | <0.001 |
| dsbA vs. dsbA_dsbB | 412.129 | 7.038 | <0.001 |
| sppA vs. dsbB | 384.915 | 6.573 | <0.001 |
| sppA vs. RV308 WT | 327.963 | 5.6 | <0.001 |
| dsbA vs. degP_skp | 317.05 | 5.414 | 0.001 |
| degP_sppA vs. dsbB | 312.45 | 5.336 | 0.001 |
| sppA_skp vs. dsbB | 312.073 | 5.329 | 0.001 |
| sppA vs. dsbA_dsbB | 305.045 | 5.209 | 0.002 |
| dsbA vs. skp | 301.523 | 5.149 | 0.002 |
| degP vs. dsbB | 263.406 | 4.498 | 0.008 |
| degP_sppA vs. RV308 WT | 255.498 | 4.363 | 0.01 |
| sppA_skp vs. RV308 WT | 255.121 | 4.357 | 0.01 |
| degP_sppA vs. dsbA_dsbB | 232.58 | 3.972 | 0.024 |
| sppA_skp vs. dsbA_dsbB | 232.203 | 3.965 | 0.023 |
| dsbA vs. degP | 228.593 | 3.904 | 0.026 |
| **OmpA-GM-CSF producing strains:** | | | |
| sppA vs. RV308 WT | 10.271 | 8.951 | <0.001 |
| sppA vs. dsbA_dsbB | 9.472 | 8.254 | <0.001 |
| sppA vs. degP_skp | 9.18 | 8 | <0.001 |
| sppA vs. sppA_skp | 7.23 | 6.3 | <0.001 |
| degP vs. RV308 WT | 6.635 | 5.782 | <0.001 |
| sppA vs. degP_sppA | 6.123 | 5.336 | 0.001 |
| degP vs. dsbA_dsbB | 5.836 | 5.085 | 0.002 |
| sppA vs. dsbA | 5.58 | 4.862 | 0.004 |
| dsbB vs. RV308 WT | 5.579 | 4.862 | 0.003 |
| degP vs. degP_skp | 5.544 | 4.831 | 0.004 |
| skp vs. RV308 WT | 5.439 | 4.74 | 0.004 |
| sppA vs. skp | 4.832 | 4.211 | 0.014 |
| dsbB vs. dsbA_dsbB | 4.78 | 4.165 | 0.016 |
| sppA vs. dsbB | 4.692 | 4.089 | 0.018 |
| dsbA vs. RV308 WT | 4.692 | 4.088 | 0.018 |
| skp vs. dsbA_dsbB | 4.64 | 4.043 | 0.019 |
| dsbB vs. degP_skp | 4.488 | 3.911 | 0.025 |
| skp vs. degP_skp | 4.348 | 3.789 | 0.032 |
| degP_sppA vs. RV308 WT | 4.149 | 3.615 | 0.046 |

**
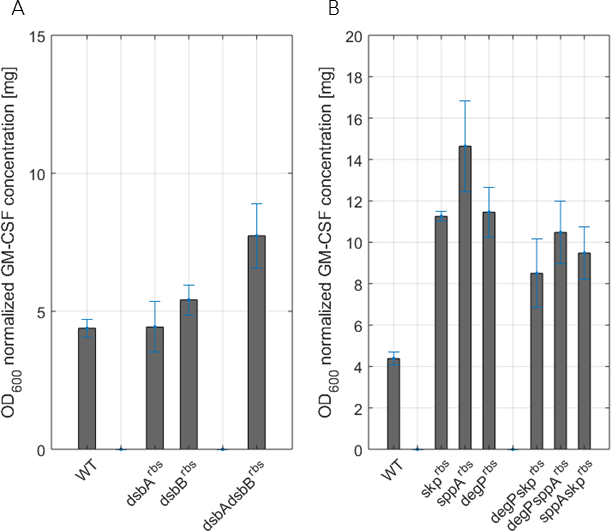
**

**Figure S1 The GM-CSF data calculated per OD_600_ unit.** Comparison of the wild-type E. coli strain RV308 and mutant strains with regulated translation rate of components of the disulfide bond formation mechanism (A) and of Skp (folding chaperone), SppA (signal peptidase), and DegP (periplasmic protease) (B).

**Table S2 Oligonucleotides used in this study**

| **Name** | **Sequence (5’ → 3’)** |
| --- | --- |
| 1. PCR primers: | |
| Gibson_ pMAZ-SK backbone_F | GTTTTAGAGCTAGAAATAGCAAG |
| Gibson_ pMAZ-SK backbone_R | GTGCTCAGTATCTCTATCACTGATAGG |
| dsbA_colony_F | TTAGGGCGTATGCACCAG |
| dsbA_colony_R | AACAAACATCGCCGGAAC |
| dsbA_seq_F | TGAGGACGCTACACTGATAC |
| dsbB_colony_F | CGTTATACAACGCGAAGTTCTG |
| dsbB_colony_R | CTGTCATCCATTTCGGATAACG |
| dsbB_seq_F | CTTTAAACGGCTGGGAAATC |
| skp_colony_F | TACGTCTAACACTTAGGGGG |
| skp_colony_R | GTAAGCAACGGCGTTTGCAT |
| skp_seq_F | CGGTTCGTACTTCCTTCTTCTG |
| sppA_colony_F | ATTATACCTAACGAGGAGAAAC |
| sppA_colony_R | TTTGCCGATGTACTGCATAG |
| sppA_seq_F | CGGGACGTTAATCGACGTAG |
| degP_colony_F | TTGAGTAACGCCACTGATCG |
| degP_colony_R | ATCAATGATGACGCCGGAAC |
| degP_seq_F | TTTCGCCGGAACGTTTAATC |
| 1. CRMAGE oligos^a^: | |
| MAGE_dsbA | aacgctaaaactaaaccagccagcgcTagccaaatctttttcatAATAAGCCTCCTTAGTTgatctactctctccgattaatacataggtgttaattgcaaagggggttc |
| MAGE_dsbB | ttggtttaaactgcgcactctatgcatattgcagggaaatgattGCGCACACCGAAGTTGGAATACGATTGatgttgcgatttttgaaTcaatgttcacaaggccggggc |
| MAGE_skp | gcccatgttgacgattgcaattttgtcagccgcctgagcagaagttgccagtgctaaaccgagacctgcagctaataaccactttttCATAATATTCCCCCTAAGTGTTAGACGTATATACTGccggagggtgcagttctttgcgtggcccggcgatcttatattgatcgcctaaagtcatcgctacactaccactacattcctttg |
| MAGE_sppA | actcctgtcgtgatatttattcacaaaattaacacgagagtggattttgttacagcacagtccgcaattcctgctgacaagtaatctattCAAACGTACCCCTTAATTATACCTAACGAGGAGAAACTatgcgaaccctttggcgatttattgccggattttttaaatggacgtggcgtctgctgaatttcgtccgtgaaatggtacttaacctgttc |
| MAGE_degP | agaagtctcagccgccgttgcagagagcggagataacgccaaacctaaactcagagcTagtgcactcagtgctaatgtggtttttttCACTTCCTCAATTTCGATCAGTGGCGTTACTCaaaattgctgtgttcttcagattcgttttatagcctgaagttccgaactaaagtttctgcaaaaggtaaaaatttattgttcgtctttac |
| 1. gRNAs with overlaps^b^: | |
| gRNA_dsbA_F | gtgatagagatactgagcacGATCATGAAAAAGATTTGGCgttttagagctagaaatagc |
| gRNA_dsbA_R | gctatttctagctctaaaacGCCAAATCTTTTTCATGATCgtgctcagtatctctatcac |
| gRNA_dsbB_F | gtgatagagatactgagcacCGCCCCGGCCTTGTGAACATgttttagagctagaaatagc |
| gRNA_dsbB_R | gctatttctagctctaaaacATGTTCACAAGGCCGGGGCGgtgctcagtatctctatcac |
| gRNA_skp_F | gtgatagagatactgagcacCCGGTGCAAATGGGATGGTAgttttagagctagaaatagc |
| gRNA_skp_R | gctatttctagctctaaaacTACCATCCCATTTGCACCGGgtgctcagtatctctatcac |
| gRNA_sppA_F | gtgatagagatactgagcacTAATCTATTGCGCCTGTGACgttttagagctagaaatagc |
| gRNA_sppA_R | gctatttctagctctaaaacGTCACAGGCGCAATAGATTAgtgctcagtatctctatcac |
| gRNA_degP_F | gtgatagagatactgagcacCCGGTGCAAATGGGATGGTAgttttagagctagaaatagc |
| gRNA_degP_R | gctatttctagctctaaaacTACCATCCCATTTGCACCGGgtgctcagtatctctatcac |

^a^ Secondary mutation introduced to disrupt PAM motif is underlined. Capital letters indicate RBS regions.

^b^ Capital letters indicate gRNA regions.

**Table S3 DNA sequences of the genes encoding the model proteins used in this study**

| Sequence of PelB-scFv173-2-5-AP: |
| --- |
| ATGAAATACCTATTGCCTACGGCAGCCGCTGGCTTGCTGCTGCTGGCAGCTCAGCCGGCCATGGCCGAGGTGCAGCTGTTGGAGTCCGGGGGAGGCTTAGTTCAGCCTGGGGGGTCCCTGAGACTCTCCTGTGCAGCCTCTGGATTCACCTTTAGCAGCTATGCCATGAGCTGGGTCCGCCAGGCTCCAGGGAAGGGGCTGGAGTGGGTCTCAGCTATTAGTGGTAGTGGTGGTAGTACATACTACGCAGACTCCGTGAAGGGCCGGTTCACCATCTCCAGAGACAATTCCAAGAACACGCTGTATCTGCAAATGAACAGCCTGAGAGCCGAGGACACGGCCGTATATTACTGTGCGAGGGGGGGAGGAGTGGTCGAATTTTGGGGCCAGGGAACCCTGGTCACTGTCTCCTCAAAGCTTTCAGGGAGTGCATCCGCCCCAAAACTTGAAGAAGGTGAATTTTCAGAAGCACGCGTAGACATCCGGATGACCCAGTCTCCATCCTTCCTGTCTGCATCTGTAGGAGACAGAGTCACCATCACTTGCCGGGCCAGTCAGGACATTAGCAGTTATTTCGCCTGGTATCAGCAAAAACCAGGGAAAGCCCCTAAGCTCCTGATCTATGCTGCATCCACTTTGCGAAGTGGGGTCCCATCAAGGTTCAGCGGCAGTGGATCTGGGACAGATTTCACTCTCACCATCAGCAGTCTGCAACCTGAAGATTTTGCAACTTACTACTGTCAACAGAGTTACAGTACCCCTCGGATCACCTTCGGCCAAGGGACACGACTGGAGATTAAAGCGGCCGCAGCCCGGGCACCAGAAATGCCTGTTCTGGAAAACCGGGCTGCTCAGGGCGATATTACTGCACCCGGCGGTGCTCGCCGTTTAACGGGTGATCAGACTGCCGCTCTGCGTGATTCTCTTAGCGATAAACCTGCAAAAAATATTATTTTGCTGATTGGCGATGGGATGGGGGACTCGGAAATTACTGCCGCACGTAATTATGCCGAAGGTGCGGGCGGCTTTTTTAAAGGTATAGATGCCTTACCGCTTACCGGGCAATACACTCACTATGCGCTGAATAAAAAAACCGGCAAACCGGACTACGTCACCTCCTCGGCTGCGTCAGCAACCGCCTGGTCAACCGGTGTCAAAACCTATAACGGCGCGCTGGGCGTCGATATTCACGAAAAAGATCACCCAACGATTCTGGAAATGGCAAAAGCCGCAGGTCTGGCGACCGGTAACGTTTCTACCGCAGAGTTGCAGGATGCCACGCCCGCTGCGCTGGTGGCACATGTGACCTCGCGCAAATGCTACGGTCCGAGCGCGACCAGTGAAAAATGTCCGGGTAACGCTCTGGAAAAAGGCGGAAAAGGATCGATTACCGAACAGCTGCTTAACGCTCGTGCCGACGTTACGCTTGGCGGCGGCGCAAAAACCTTTGCTGAAACGGCAACCGCTGGTGAATGGCAGGGAAAAACGCTGCGTGAACAGGCACAGGCGCGTGGTTATCAGTTGGTGAGCGATGCTGCCTCACTGAATTCGGTGACGGAAGCGAATCAGCAAAAACCCCTGCTTGGCCTGTTTGCTGACGGCAATATGCCAGTGCGCTGGCTAGGACCGAAAGCAACGTACCACGGCAATATCGATAAGCCCGCAGTCACCTGTACGCCAAATCCGCAACGTAATGACAGTGTACCAACCCTGGCGCAGATGACCGACAAAGCCATTGAATTGTTGAGTAAAAATGAGAAAGGCTTTTTCCTGCAAGTTGAAGGTGCGTCAATCGATAAACAGGATCATGCTGCGAATCCTTGTGGGCAAATTGGCGAGACGGTCGATCTCGATGAAGCCGTACAACGGGCGCTGGAATTCGCTAAAAAGGAGGGTAACACGCTGGTCATAGTCACCGCTGATCACGCCCACGCCAGCCAGATTGTTGCGCCGGATACCAAAGCTCCGGGCCTCACCCAGGCGCTAAATACCAAAGATGGCGCAGTGATGGTGATGAGTTACGGGAACTCCGAAGAGGATTCACAAGAACATACCGGCAGTCAGTTGCGTATTGCGGCGTATGGCCCGCATGCCGCCAATGTTGTTGGACTGACCGACCAGACCGATCTCTTCTACACCATGAAAGCCGCTCTGGGGCTGAAACTGGGGGATATCGCA  *alpl* (AP)  *scFv173*  *pelB*  *alpl* (AP) |
| Sequence of OmpA-GM-CSF: |
| ATGAAAAAAACTGCTATCGCTATCGCTGTTGCTCTGGCTGGTTTCGCTACTGTTGCTCAGGCGGCGGCCATGGCTGCACCCGCCCGCTCGCCCAGCCCCAGCACGCAGCCCTGGGAGCATGTGAATGCCATCCAGGAGGCCCGGCGTCTCCTGAACCTGAGTAGAGACACTGCTGCTGAGATGAATGAAACAGTAGAAGTCATCTCAGAAATGTTTGACCTCCAGGAGCCGACCTGCCTACAGACCCGCCTGGAGCTGTACAAGCAGGGCCTGCGGGGCAGCCTCACCAAGCTCAAGGGCCCCTTGACCATGATGGCCAGCCACTACAAGCAGCACTGCCCTCCAACCCCGGAAACTTCCTGTGCAACCCAGATTATCACCTTTGAAAGTTTCAAAGAGAACCTGAAGGACTTTCTGCTTGTCATCCCCTTTGACTGCTGGGAGCCAGTCCAGGAGG  *gm-csf*  *ompA* |


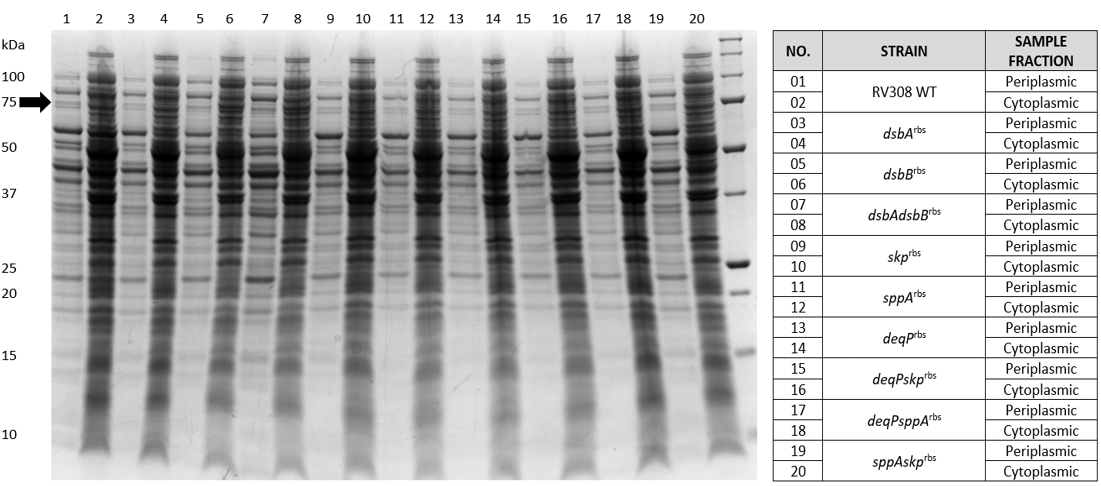


**Figure S2 SDS-PAGE of reduced periplasmic and cytoplasmic samples (10x diluted) of scFv173-2-5-AP producing strains.** The arrow indicates the size of the relevant model protein band.


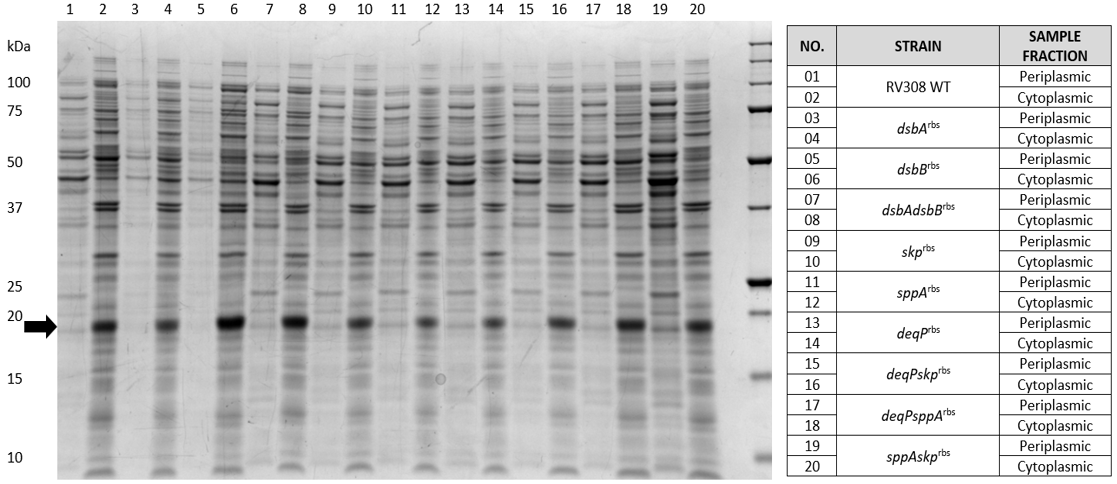


**Figure S3 SDS-PAGE of reduced periplasmic and cytoplasmic samples (10x diluted) of GM-CSF producing strains.** The arrow indicates the size of the relevant model protein band.
